# Interpenetrating Polymer Network Hydrogel Composition Alters Encapsulated MSC Spreading and In Vivo Degradation Behavior

**DOI:** 10.1021/acsbiomaterials.5c00980

**Published:** 2025-08-04

**Authors:** Liaura Ifergan-Azriel, Orit Bar-Am, Galit Saar, Talia Cohen, Claudia Loebel, Jason A. Burdick, Dror Seliktar

**Affiliations:** † The Faculty of Biomedical Engineering, Technion-Israel Institute of Technology, Haifa 3200003, Israel; ‡ The Bruce Rappaport Faculty of Medicine, Technion−Israel Institute of Technology, 1 Efron St., Haifa 3109601, Israel; § Materials Science & Biomedical Engineering Department, University of Michigan, Ann Arbor, Michigan 48109, United States; ∥ BioFrontiers Institute and Department of Chemical and Biological Engineering, University of Colorado, Boulder, Colorado 80303, United States

**Keywords:** biomaterials, tissue engineering, hydrogels, scaffolds, stem cells, interpenetrating polymer network

## Abstract

An interpenetrating polymer network (IPN) hydrogel was developed for the three-dimensional (3D) culture of multipotent mesenchymal stromal cells (MSCs) with the aim of independently controlling cell spreading and material modulus. Based on our previous studies, we formulated a semisynthetic material composed of two networks: a covalent network of poly­(ethylene glycol) (PEG)-fibrinogen (PF) and a second guest–host (GH) network of hyaluronic acid (HA) coupled to β-cyclodextrin (CD) and adamantane (Ad). The PF network provided cell attachment, precise control over modulus through the incorporation of additional PEG-diacrylate (PEG-DA) cross-linking, and proteolytic degradability. The GH-HA network contributed to the hydrogel’s dynamic properties through enhanced viscoelasticity. This dynamic versatility enabled MSCs to better spread and grow in the IPN, even within highly cross-linked formulations. We also observed that the IPN facilitated significantly faster cell spreading kinetics, independent of the material modulus, when compared to single-network PF hydrogels. Hydrogel biodegradation was also characterized after subcutaneous implantation for up to 8 weeks by using MRI analysis. Increasing the PEG-DA cross-linking of the IPN significantly accelerated the in vivo bioresorption, whereas the biodegradation in single-network PF hydrogels was significantly delayed by the additional PEG-DA. We conclude that the covalent cross-links maintain the bulk structural integrity of the hydrogel, whereas the reversible GH interactions provide more localized adaptability for cell-mediated proteolysis and matrix remodeling, possibly through increased network heterogeneity. This design effectively mimics the ECM by providing a more supportive environment for encapsulated cells that allows them to adhere, spread, and proliferate, which may be useful in various MSC-based tissue engineering and regenerative medicine applications.

## Graphical abstract

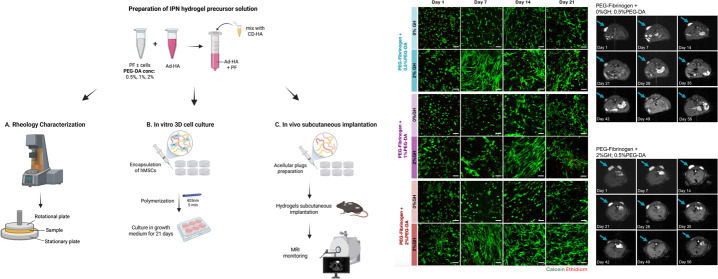

## Introduction

Over the last two decades, significant advancements have been made in the field of tissue engineering and regenerative medicine with the aid of new discoveries in stem cell research. One key area of research focus has been cell sourcing, with multipotent mesenchymal stromal cells (MSCs) emerging as an abundant alternative to fully differentiated cell lineages due to their self-renewal capability.^1–3^ Another advantage of MSCs is their multipotency as they have the potential to differentiate into various cell types, including those found in bone, cartilage, fat, muscle, and connective tissue cells.^4^ This multipotency allows MSCs to contribute to the regeneration of different tissue types and promotes the formation of functional tissues after implantation.^5^ In addition to their regenerative potential, MSCs also secrete various bioactive molecules including growth factors, cytokines, and extracellular vesicles. These paracrine factors have also been shown to promote tissue repair.^6,7^

Despite the many advances of MSCs in regenerative medicine, it is becoming increasingly evident that they may not be able to facilitate tissue repair alone and may require a biomaterial scaffold to achieve their full potential.^8^ Indeed, biomaterials have been shown to play a crucial role in localizing the effects of MSCs in tissue repair as well as in controlling cellular functions including cell adhesion, proliferation, and differentiation in muscle, nerve, and cartilage repair.^9–12^ Engineering biomaterial scaffolds to support and guide the growth and development of MSCs toward specific tissue types remains a formidable challenge.^13,14^ Extensive research efforts are now focused on developing biomaterials that provide an optimal microenvironment for MSCs, promoting their survival, differentiation, morphogenesis, and tissue maturation.^15,16^

Among the various biomaterial options available, hydrogels have gained considerable attention as leading candidates for scaffolds in tissue repair applications.^17–19^ Hydrogels used in a biomedical context are typically categorized as either synthetic or natural materials.^20^ Among synthetic polymers, poly­(ethylene glycol) (PEG) has been extensively studied for its biocompatibility and tunable mechanical and chemical properties.^21^ However, PEG hydrogels lack sufficient bioactivity to control cell adhesion, proliferation, or differentiation of resident MSCs.^22^ To address these limitations, modifications to PEG have been introduced to endow these hydrogels with specific activity,^10^ including cell adhesion and proteolytic degradability.^23^ We have previously modified PEG with fibrinogen through a covalent reaction between PEG-diacrylate (PEG-DA) and cysteines found on fibrinogen.^24^ The PEG-fibrinogen (PF) adducts provide cell adhesion motifs and proteolytic degradability to the PF hydrogels.^25^ These semisynthetic PF hydrogels have demonstrated good potential for cell encapsulation and tissue repair applications.^26–28^ Nevertheless, the PF hydrogel as a single network (SN) has its limitations, particularly in a mechanical context. The PF hydrogels are soft materials with shear storage moduli ranging between 100 and 500 Pa.^29^ Although the modulus can be easily and considerably increased by adding PEG-DA cross-linker to the hydrogel precursor solution during gelation, this alteration also reduces the hydrogel’s susceptibility to proteolysis and therefore comes at the expense of impeding cell-mediated matrix remodeling.^30^ Thus, without nanostructural modifications, the cell spreading and cell motility may be hindered with increasing modulus of the PF hydrogel.^31,32^

The ability to enhance cell-mediated remodeling in a stiff encapsulating matrix is a challenge that, if achieved, may provide even greater versatility to guide MSCs toward natural tissue repair using semisynthetic hydrogel scaffolds. In vivo, MSCs often encounter stiff matrix properties of the natural ECM that do not impede motility and remodeling. This decoupling occurs naturally owing to the properties of the ECM, namely, the unique multicomponent composition of the natural ECM matrix. To achieve a similar design in hydrogels, one could consider the use of composite materials in the form of interpenetrating polymer network (IPN) hydrogels.^33^ An IPN hydrogel consists of two or more polymer networks that are intertwined and interlocked on a molecular level.^34^ An IPN hydrogel provides more control over mechanical properties, as compared to SN hydrogels, namely, through compositional modification of each respective polymer network.^35^ IPN hydrogels can also support 3D cell culture, although compositional design considerations are required to facilitate cell spreading within the polymer network structure.^36^ Such cell-compatible IPN hydrogels have recently been explored as scaffolds for tissue engineering. These materials can be made with biological polymers,^37–39^ synthetic polymers,^36^ or a combination of both.^34,40,41^ Biological IPN hydrogels provide inherent bioactivity for cell adhesion,^37^ whereas synthetic IPN hydrogels require further modifications with bioactive ligands to enable cell attachment.^36^ Semisynthetic IPN hydrogels can achieve both bioactivity and structural versatility through the compositional design of the polymer systems. Hence, a semisynthetic IPN can be designed to be very stiff and still provide support for encapsulated cells to grow and remodel within the matrix,^34,42^ essentially mimicking the natural cellular microenvironment of the ECM.

Considering that matrix stiffness is one of the critical factors influencing 3D cell differentiation and tissue morphogenesis,^43^ IPN hydrogels are a particularly promising choice for developing ECM analogs in load-bearing tissue engineering applications where conventional SN hydrogels are inadequate.^44,45^ In this context, we have previously reported on a semisynthetic IPN hydrogel composed of two networks: a covalently cross-linked network made of PF and a second guest–host (GH) network made from hyaluronic acid (HA) coupled to β-cyclodextrin (CD) and adamantane (Ad).^46^ The PF network provides several important features, including cell attachment, controllable modulus through the incorporation of additional PEG-DA cross-linking, bioactivity, and proteolytic degradability.^47–50^ The covalent cross-links maintain the structural integrity of the hydrogel and provide versatility in terms of mechanical stability. The GH-HA network contributes to the hydrogel’s dynamic properties through viscoelasticity. Unlike the covalent cross-links, GH interactions are reversible, allowing for flexibility and adaptability within the hydrogel structure.^51^ This dynamic versatility and mimicking of the natural ECM enables cell spreading, even within highly cross-linked PF formulations. In this study, we performed a thorough characterization of material viscoelastic properties and then investigated the influence of these properties on MSC spreading when encapsulated in 3D hydrogels, as well as degradation behavior in vivo. Ultimately, the controllable modulus and dynamic properties of this IPN hydrogel make it a promising candidate in various tissue engineering and regenerative medicine applications using MSCs.

## Methods

### Material Synthesis

Tetrabutylammonium salt of hyaluronic acid (HA-TBA) was prepared using HA (75 kDa; Lifecore Biomedical) and ion exchange (Dowex 50Wx8 hydrogen form) followed by neutralization with aqueous tetrabutylammonium hydroxide (0.4 M) as described earlier.^46,51^ The HA-TBA was then further reacted with 1-adamantane acetic acid (Ad) to form the Ad-HA. Similarly, HA-TBA was reacted with 6-(6-aminohexyl)­amino-6-deoxy-β-cyclodextrin (CD) to form CD-HA by anhydrous reaction in DMSO as described previously.^46,51^ The Ad-HA and CD-HA were dialyzed against phosphate-buffered saline (PBS) and stored at room temperature after lyophilization. The PEG-fibrinogen (PF) was prepared by reacting bovine fibrinogen (Seqens In vitro Diagnostics, France) with linear PEG-diacrylate (PEG-DA, 10 kDa) under reducing conditions as detailed elsewhere.^52^ The PF product was precipitated in acetone and dialyzed against PBS. The AD-HA, CD-HA, and PF were characterized by ^1^H NMR (Bruker 360 MHz) prior to use.^53^

### Hydrogel Formation

IPN hydrogels were made with 8 mg/mL PEG-fibrinogen containing 2% (w/v) GH concentration with Ad-HA (29% modification) and CD-HA (25% modification) and a 1:1 ratio of adamantane and β-cyclodextrin. The hydrogels were prepared with two solutions of 8 mg/mL PEG-fibrinogen (PF) in PBS containing Ad-HA and CD-HA, respectively. A photoinitiator (0.01% w/v lithium phenyl (2,4,6-trimethylbenzoyl) phosphinate, LAP, Sigma) and additional PEG-DA (0.5, 1, 2% w/v) were also added. The two solutions were mixed, centrifuged briefly, and cast into cylindrical constructs by exposure to 405 nm blue light (2.2 mW/cm^2^). Control single-network hydrogels were made from 8 mg/mL PF containing 0.5, 1, or 2% w/v additional PEG-DA and photoinitiator (0.01% w/v LAP).

### Characterization of the Hydrogel Viscoelastic Properties

Rheological properties of the IPN hydrogels, including shear storage modulus (G′) and shear loss modulus (G″), were measured using a stress-controlled rheometer (AR-G2, TA Instruments) fitted with a 20 mm diameter parallel plate geometry, at room temperature, with 2% strain at an angular frequency of 3 rad/s. PF precursor solution (with a fibrinogen concentration of 8 mg/mL) containing varying amounts of PEG-DA was mixed with 8 mg/mL PF containing different concentrations of Ad-HA guest and CD-HA host (illustrated in Figure 1). The cell-laden IPN precursor solution was supplemented with 0.01% (w/v) LAP photoinitiator (Sigma) and covalently cross-linked by exposure to blue light (405 nm, 2.2 mW/cm^2^) for 90 s. Hydrogel rheological properties were monitored with light exposure using a light guide attachment capable of transmitting the blue light to the sample during the time-sweep measurement. The light illumination was applied 15 s after the start of the rheological measurements and the shear modulus values were continuously recorded until the shear storage modulus reached its plateau value (approximately 90 s).

**Figure 1:**
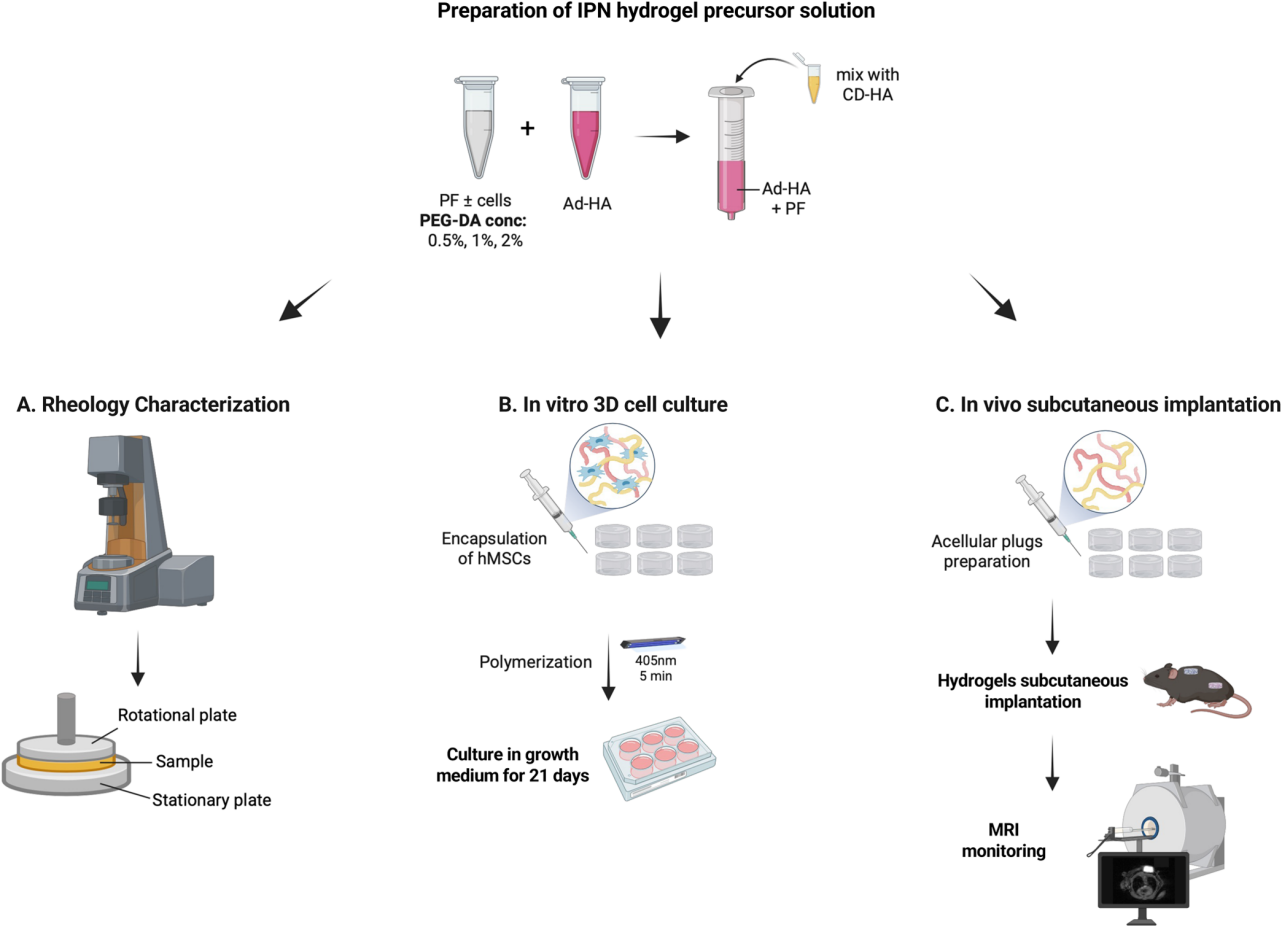
Schematic illustration of the experimental design for testing the IPN hydrogels. A PF precursor solution containing LAP photoinitiator, with or without cells (hMSCs), is supplemented with additional PEG-DA cross-linker, and separately mixed with Ad-HA (guest) and CD-HA (host) to form two PF-HA precursor solutions. The two solutions are pipetted together to form the final IPN precursor solution. The solution is then exposed to blue light (405 nm, 2.2 mW/cm^2^) for up to 5 min to facilitate the covalent cross-linking reaction. Rheological testing is performed using an acellular solution with a specialized in situ light guide geometry assembly on the rheometer. Cell viability and morphogenesis studies are performed on cell-seeded plug constructs prepared in 5 mm cylindical molds. The constructs are subsequently grown in culture medium for up to 3 weeks. In vivo implantation and biodegradation studies are performed on acellular plug constructs made in 5 mm cylindrical models with gadolinium-labeled hydrogel precursors. The labeled gadolinium-hydrogel constructs are implanted subcutaneously on the backs of mice and tracked by MRI analysis for up to 8 weeks.

### Human Mesenchymal Stromal Cell Culture

Bone marrow-derived human mesenchymal stromal cells (hMSCs) (Lonza, Switzerland) were expanded in 75 cm^2^ tissue culture flasks under standard culture conditions in MSC NutriStem defined, xeno-free, and serum-free basal medium, containing 30 μg/mL gentamycin sulfate and 15 ng/mL amphotericin B solution (Biological Industries, Israel). Flasks were incubated in a water-jacketed CO_2_ incubator (37 °C, 5% CO_2_). Cells were harvested using trypsin EDTA solution B (Biological Industries), centrifuged at 1250 rpm for 5 min, and encapsulated within hydrogels or subcultured for future experiments.

### Cell Encapsulation in Hydrogels and Cell Viability Analysis

Hydrogels were prepared as illustrated in Figure 1. For IPN hydrogels, PF precursor solution containing PF and varying amounts of PEG-DA was used to suspend the cell pellet after trypsinization and centrifugation of the cells. The PF and cells were then mixed with PF containing the Ad-HA guest and CD-HA host separately. The cell-laden IPN precursor solution was supplemented with 0.01% (w/v) LAP photoinitiator and placed in a cylindrical mold (5 mm diameter, 5 mm height). The solution was then covalently cross-linked by exposure to blue light (405 nm, 2.2 mW/cm^2^) for 90 s. The hydrogels seeded with hMSCs were then removed from the molds and placed in MSC growth medium. The cell seeding density used in all experiments was 1 × 10^6^ cells/mL of hydrogel precursor solution. The encapsulated cells in the hydrogels were maintained in culture for 3 weeks and analyzed at several time points (days 1, 7, 14, and 21). A Live/Dead assay was performed according to published protocols whereby cells were stained with 4 mM calcein-AM solution and 2 mM ethidium homodimer I solution (in DMSO, Sigma) and imaged using a Zeiss LSM700 confocal microscope.^54^ Quantitative viability was determined by liberating the cells from the hydrogels using a gel digestion assay with 0.5 mg/mL collagenase (Sigma) for 2 h at 37 °C as described elsewhere.^25^ The live/dead cell counting was performed using the Countess automatic cell counter (Invitrogen) with trypan blue staining (0.4%, Invitrogen)

### Cell Spreading Analysis and Image Processing

Calcein-AM images obtained at each time point (*n* = 3) were processed using a MATLAB (MathWorks) code that performs image processing operations on a selected TIFF image file. The image was preprocessed by enhancing contrast and removing noise. A binary image was generated using adaptive thresholding followed by morphological operations and watershed segmentation to extract and analyze cell morphology. The algorithm then calculated the aspect ratio of each segmented cell within the image. In addition, circular cells were identified based on predetermined criteria, and their boundaries outlined accordingly (see Figure S1). The code provides a visual representation of the segmented cells and the original grayscale image, allowing confirmation of the validity of the processing. The areas of the rounded cells and spread cells were calculated, and the percentage of cell spread area was determined according to the calculations provided in Figure S1.

### In Vivo Study: MRI Assessment of Hydrogel Integration/Degradation

All animal studies were approved by the Animal Board and Safety Committee of the Technion. Adult male C57BL/6 mice were anesthetized with ketamine (80 mg/kg) and xylazine (15 mg/kg). Buprenorphine (0.02 mg/kg) and lidocaine (0.5%) were injected for analgesia and local anesthesia, respectively. One dorsal subcutaneous pocket was formed over each shoulder and hip (A: left shoulder; B, right shoulder; C: middle hip region) for a total of three pockets per animal. A single plug was implanted in each pocket, and the incisions were either sutured or glued. During the first week, animals were checked daily to monitor wound healing at the incision site. The integration and degradation of the hydrogels implanted subcutaneously were documented and monitored by using MRI for 8 weeks. After 8 weeks, all mice were humanely sacrificed and biopsies were taken from the implantation sites.

Calibration of the concentration of contrast agent Gd-DTPA required for MRI monitoring was performed in vitro with increasing concentrations of PF-Gd-DTPA (0 to 3 mg/mL, Figure S2). A concentration of 2.5 mg/mL PF-Gd-DTPA was chosen to be incorporated in the PF hydrogel precursor solution (with a final concentration of 8 mg/mL) containing varying amounts of PEG-DA (0.5, 1, 2%) and GH-HA (0 or 2%). A total of four replicates per formulation were prepared with an initial volume of 50 μL each. Prior to implantation, the samples were placed in sterile phosphate-buffered saline (PBS) solution overnight at 4 °C. The gadolinium (Gd) signal intensity was quantified using a compact animal scanner at 1T (Aspect M2, Aspect Imaging). The in vivo MR images were acquired in the same system with animals inserted into the scanner under a continuous flow of 0.5–1.5% isoflurane, supplemented with O_2_ (0.8 L/min). They were monitored using T1-weighted sequence protocols: GRE-SP (Gradient Echo) sequence with slice thickness 1/4 = 1 mm, FOV 1/4 = 6.4 × 6.4 cm, matrix dimension 1/4 = 128 × 128, repetition time TR 1/4 = 12.6 ms, echo time TE 1/4 = 3.2 ms, flip angle 1/4 30°. T1-map images were calculated by performing exponential curve fitting for each pixel using custom-built software in MATLAB (MathWorks).

### Histological Analysis

Full-thickness subcutaneous samples consisting of skin and muscle were excised after 8 weeks. Samples were fixed in 4% paraformaldehyde (v/v in PBS, Santa Cruz Biotechnology) for 1 day and embedded in paraffin, according to standard protocols. Samples were cut in 5 μm intervals perpendicular to the skin surface in the center of each implant. The sectioned samples were prepared for hematoxylin and eosin staining (H&E, Sigma) using standard protocols. The stained samples were digitally scanned using a Pannoramic 250 Flash III automated digital scanner (3D Histech Ltd.) using a 20*X*/0.8 Plan Apochromat objective.

### Statistics

All experiments were performed with three or four replicates as indicated in the figure legends. Statistical analysis among groups was assessed using either Student’s *t* test or two-way ANOVA with Tukey HSD post hoc testing, using GraphPad Prism 9 software. To assess statistical significance in the data, the following *p*-values were used: *p < 0.05; **p < 0.01; ***p < 0.001; ****p < 0.0001.

## Results

### Effect of PEG-DA and GH Addition to the PF Network

Single-network hydrogels were formed through the mixing of PF and PEG-DA. The concentration of PEG-DA was adjusted to control the covalent cross-linking density and mechanical properties of the PF hydrogel (Figure 2A). Upon initiation of cross-linking (black arrows) by blue light illumination (405 nm, 2.5 mW/cm^2^), the photoinitiator generates reactive species that initiate the cross-linking reaction between acrylates on the PF adducts. This reaction culminates in covalent cross-linking of the PF into a three-dimensional network. The addition of free PEG-DA molecules increases the covalent cross-linking of the PF hydrogel, thereby increasing cross-link density and resulting in a denser network structure with enhanced mechanical strength and modulus. The shear storage modulus G′ (Pa) was directly proportional to the cross-linking density (Figure 2C).^55,56^ High concentrations of PEG-DA (1, 2%) resulted in hydrogels with a modulus of G′ = 979 ± 39.3 Pa and G′ = 2530 ± 23.1 Pa, respectively, which was greater than G′ = 502 ± 53.2 Pa for hydrogels without PEG-DA. The full list of hydrogel properties obtained from the rheological analysis is summarized in Table 1. The kinetics of the gelation reaction was also dependent on the concentration of additional PEG-DA in the hydrogel precursor solution. The time to fully cross-link the polymer network was defined as the time it takes for the modulus to reach its plateau value after light exposure. For hydrogel with 0.5% PEG-DA, it took 30 s to fully cross-link the hydrogel, whereas formulations with higher concentrations of PEG-DA (i.e., 1, 2%) reached a plateau at approximately 1 min after light exposure.

**Figure 2:**
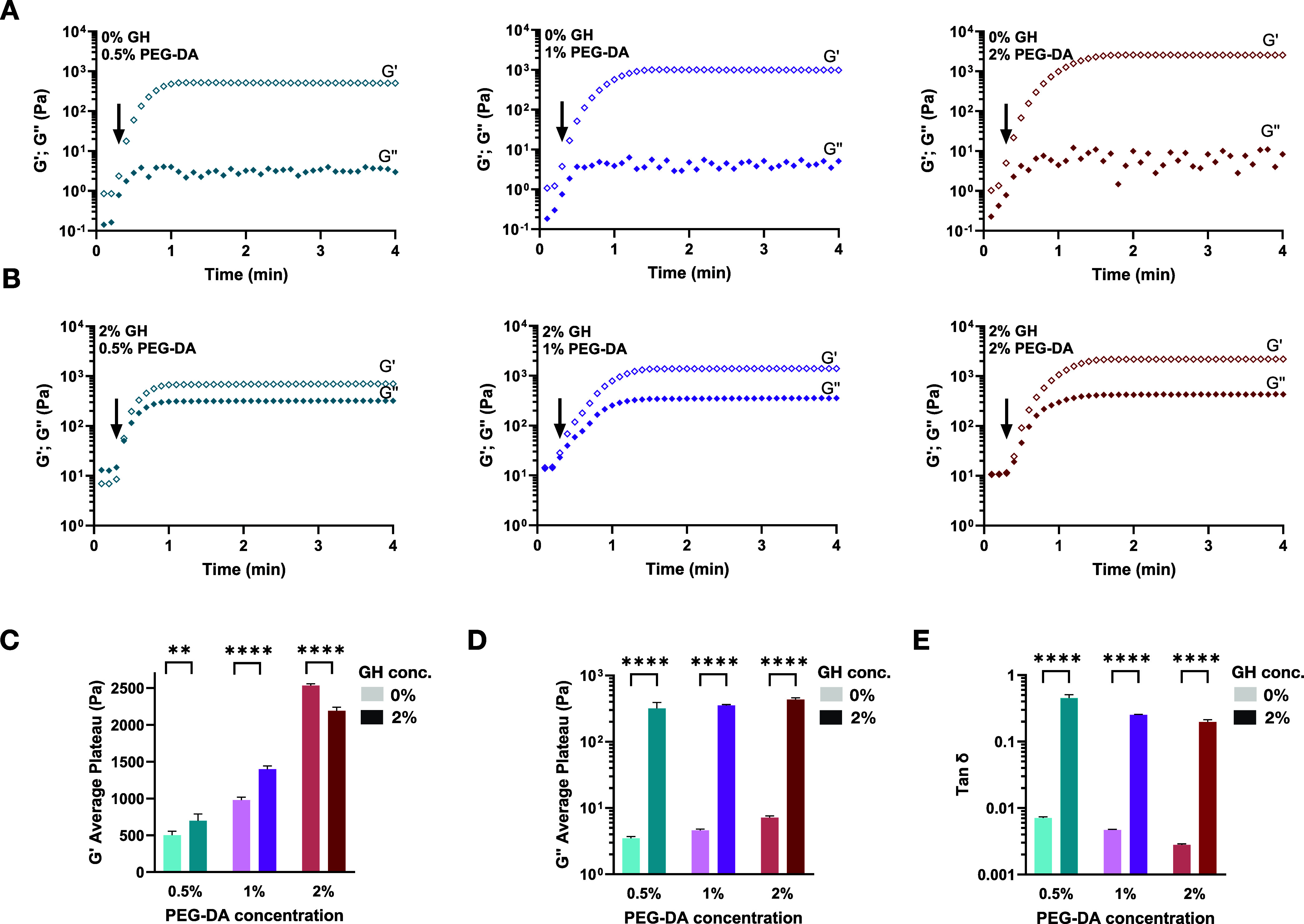
Rheological properties of IPN and single-network PF hydrogels. Formulation of several hydrogels was tested including 8 mg/mL PF with different concentrations of PEG-DA (0.5, 1, 2%) and GH-HA (0%, 2%). Oscillatory shear rheological measurements (3 rad/s, 2% strain) show the shear storage modulus G′ (Pa) and shear loss modulus G′ (Pa) as a function of time for IPN hydrogels with 2% GH-HA (B) and for hydrogels formed by PF alone (without GH-HA) for comparison (A). Light activation of covalent cross-linking was initiated after 15 s from the start of the time-sweep measurement (indicated by the arrows on the graphs). The average plateau storage modulus G′ (C), loss modulus G’ (D), and tanδ (E) of IPN and single-network PF hydrogels are summarized in the graphs (*n* = 4 replicates per group, mean ± SD, ****p < 0.0001 by two-way ANOVA with Tukey’s post hoc).

**Table 1:** Rheological Properties of Single Network and IPN Hydrogels Made with Different PED-DA Formulations

**PF+x%PEG-DA**	**0.5%PEG-DA**	**1%PEG-DA**	**2%PEG-DA**
average plateau storage modulus G′ [Pa]	502.3 ± 53.2	979.9 ± 39.3	2534.4 ± 23.1
average plateau loss modulus G″ [Pa]	3.5 ± 0.2	4.6 ± 0.2	7.2 ± 0.4
tan(δ)=G″/G′	0.0071 ± 0.0003	0.0047 ± 0.0001	0.0028 ± 0.0001

**PF+GH+x%PEG-DA**	**0.5%PEG-DA**	**1%PEG-DA**	**2%PEG-DA**
average plateau storage modulus G′ [Pa]	698.3 ± 91.5	1399.1 ± 41.7	2190.4 ± 41.7
average plateau loss modulus G″ [Pa]	319.6 ± 72.4	354.8 ± 9.3	431.8 ± 28.8
tan(δ)=G″/G′	0.4538 ± 0.0533	0.2537 ± 0/0034	0.1973 ± 0.0153

PEG-DA, as a cross-linker of the PF, did not have a significant impact on the loss modulus, G″ (Figure 2A,D). In contrast, the addition of the GH-HA network did influence the shear storage modulus and drastically increased the shear loss modulus by 2 orders of magnitude (Figure 2B–D). The observed effect was amplified as the concentration of PEG-DA increased, resulting in higher values of G″ to approximately 200 Pa for 0.5% PEG-DA and 500 Pa for 2% PEG-DA when 2% GH-HA was included. The ratio of the loss modulus (G″) to the storage modulus (G′), or tan­(δ), indicated that the addition of the GH shifted the relative dominance of the viscous response over the elastic response in the IPN system (Figure 2E). This data indicate a more viscoelastic behavior from the IPN as compared to the PF single network.

### Cell Viability

The viability of hMSCs in the PF single network and IPN hydrogel constructs containing 8 mg/mL PF with increasing concentrations of PEG-DA (0.5, 1, 2%), without or with (2%) GH-HA was confirmed for up to 3 weeks in 3D culture. Cells were mostly viable as visualized by the Live/Dead assay across different timepoints (days 1, 7, 14, and 21) and in all formulations. The viability results in IPN hydrogel formulations illustrated the cytocompatible nature of the matrix (Figure 3A). The hMSCs demonstrated a typical mesenchymal morphology and exhibited signs of cellular network formation throughout the 21-day period in all formulations. Quantitative viability data confirmed the visual observations from the calcein/ethidium staining (Figure 3B). The addition of the GH network to the PF did not have a statistically significant effect on the viability of the encapsulated cells. Overall, hMSCs in PF hydrogels with 0.5% PEG-DA maintained a viability of approximately 80% and showed no significant decrease in cell survival over the course of 21 days in 3D culture. The viability of the hMSCs when cultured in the IPN hydrogels fabricated with higher concentrations of PEG-DA was not affected at day 7; however, a reduction in the viability of hMSCs was apparent after 14 and 21 days when higher concentrations of PEG-DA were used in the formulations. This trend in reduced viability with increasing concentrations of PEG-DA was also evident in single-network PF hydrogels as reported previously^30^

**Figure 3:**
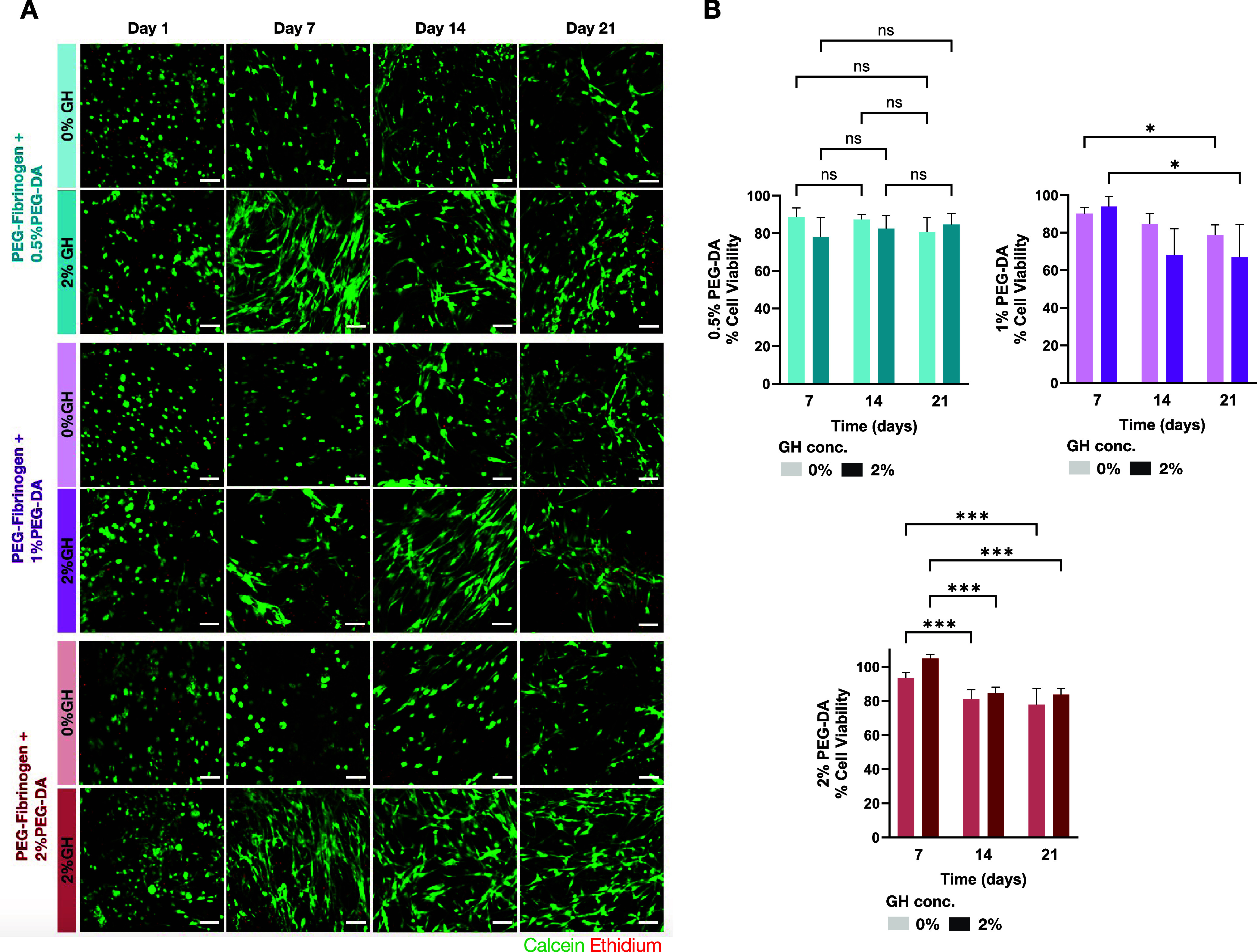
Cell viability in IPN and single-network PF hydrogels throughout 21 days of 3D culture. (A) A Live/Dead assay is used to stain live cells in calcein (green) and dead cells in ethidium (red). The results show the viability of hMSCs in the hydrogel constructs containing 8 mg/mL PF with different concentrations of PEG-DA (0.5, 1, 2%), with 2% GH-HA or without GH-HA (0%). The hMCs were three-dimensionally cultured in growth (self-renewal) medium for 21 days (scale bar = 100 μm). (B) Quantitative viability data measures the percentage of live cells in the hydrogels by liberating the cells from the hydrogels using a gel digestion assay with 0.5 mg/mL collagenase, followed by cell counting with trypan blue exclusion assay. Results are normalized to day 1 (*n* = 3 replicates per group, mean ± SD). Statistically significant differences observed between the treatments by two-way ANOVA with Tukey’s post hoc analysis are indicated (*n* = 4 replicates per group, mean ± SD, ***p < 0.001, *p < 0.05).

### Cell Morphology

Image processing of the hMSC shape within the hydrogel constructs showed a high percentage of cells that exhibited a nonrounded morphology inside the IPNs, as measured by cellular aspect ratio (Figure 4A). Initially, fewer cells exhibited a elongated morphology and remained rounded in the hydrogels, as indicated by their lower aspect ratio, particularly in the PF single-network hydrogels. As expected, adding higher concentrations of PEG-DA (i.e., 1 or 2% w/v) hindered cell spreading in the single-network PF hydrogels. The addition of the GH-HA in combination with PF and PEG-DA resulted in an immediate enhancement of the cell spreading toward network formation within the IPNs as measured by percent cell spread area, even as early as day 1 (Figure 4B). This trend was consistently observed across all time points (Figure 4B) and formulations (Figure 4C), highlighting the beneficial impact of GH-HA on the formation of cellular networks within the hydrogel. By day 14, most of the treatment groups demonstrated a convergence toward more than 80% cell spread area, with the exception of the PF + 2% PEG-DA single-network PF formulation (Figure 4C). Consequently, IPN hydrogels made with this high concentration of PEG-DA enabled cells to exhibit a statistically significant increase in their aspect ratio (Figure 4A), which likely contributed to the enhanced cell spreading area within the IPN after 2 weeks, when compared to the single-network PF treatment.

**Figure 4:**
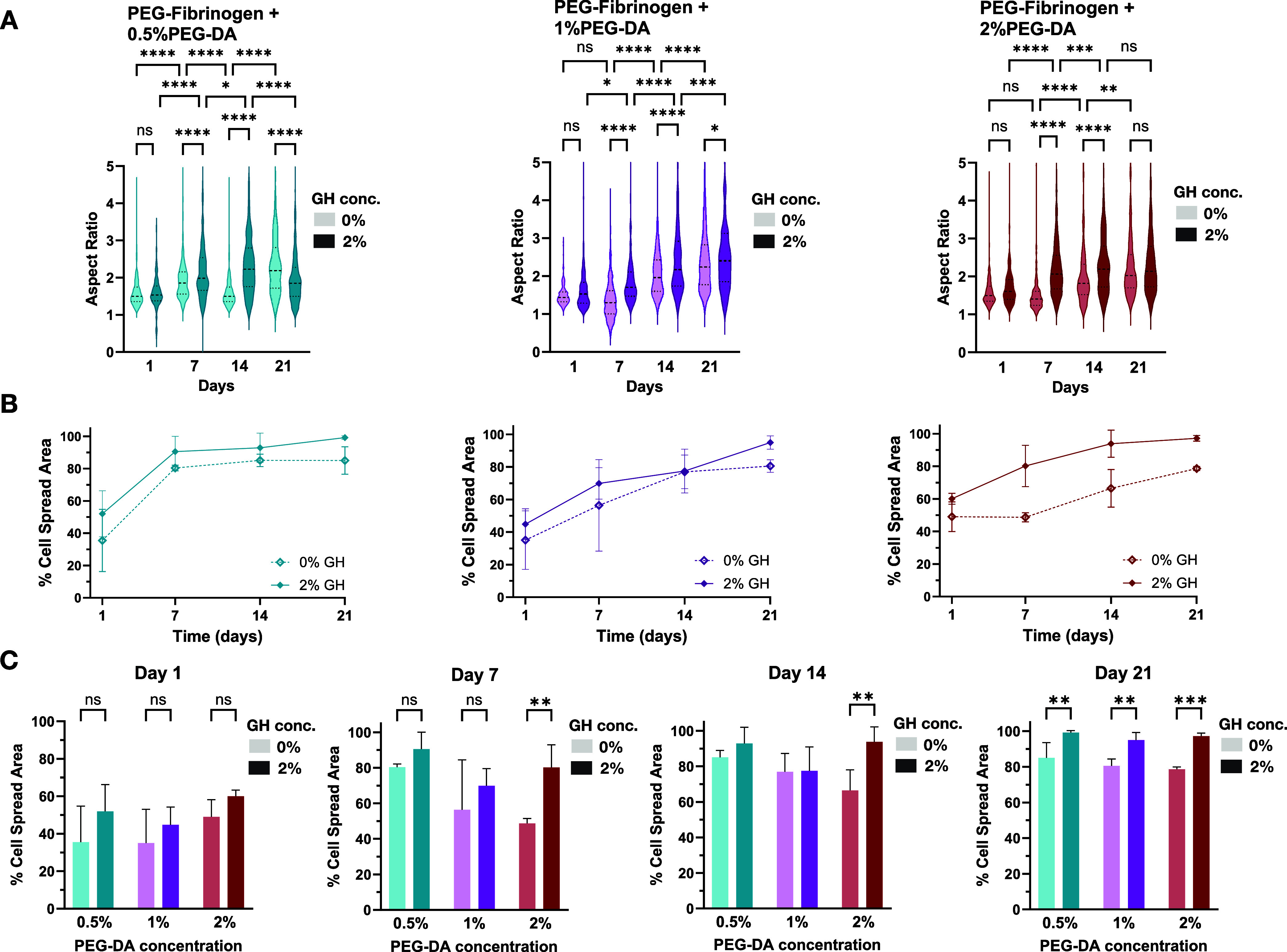
Quantitative morphometrics of cell spreading within the IPN and single-network PF hydrogels. The aspect ratio of live cells exhibiting a nonspherical morphology (i.e., cells that have spread within the matrix) was determined using an image analysis algorithm (A). The MATLAB algorithm uses confocal images of calcein-stained hydrogel samples. The percentage of nonrounded cells in the network as a function of time (B) is summarized for the different formulations at different time points. The morphometric data is also presented as a function of PEG-DA concentration (C), with or without GH, to reveal different kinetic patterns associated with the IPN’s unique properties (*n* = 3 replicates per group, mean ± SD, *p < 0.05, **p < 0.01, ***p < 0.001, ****p < 0.0001).

### In Vivo Hydrogel Biodegradation

The in vivo bioresorption process over 8 weeks was verified by MRI analysis for both the single-network PF and IPN hydrogels, which were labeled with gadolinium and subcutaneously implanted. First, the required concentration of PF-Gd-DTPA was determined based on in vitro MRI calibrations that were conducted in a 1T micro-MRI machine (Aspect M2, Aspect Imaging). The results of this calibration showed that a concentration of 2.5 mg/mL PF-Gd-DTPA (i.e., PF labeled with the contrast agent) should be combined with 5.5 mg/mL nonlabeled PF to provide sufficient contrast for quantitative MRI analysis (Figure S2). This concentration was subsequently used for all in vivo treatments, including samples of PF constructs made with varying amounts of PEG-DA (0.5, 1, and 2%) and GH-HA (0 or 2%). Images of every construct for each formulation (50 μL volume per construct, three constructs per animal) were acquired using MRI immediately after implantation and weekly for 8 weeks (Figures 5A,B and S3).

**Figure 5:**
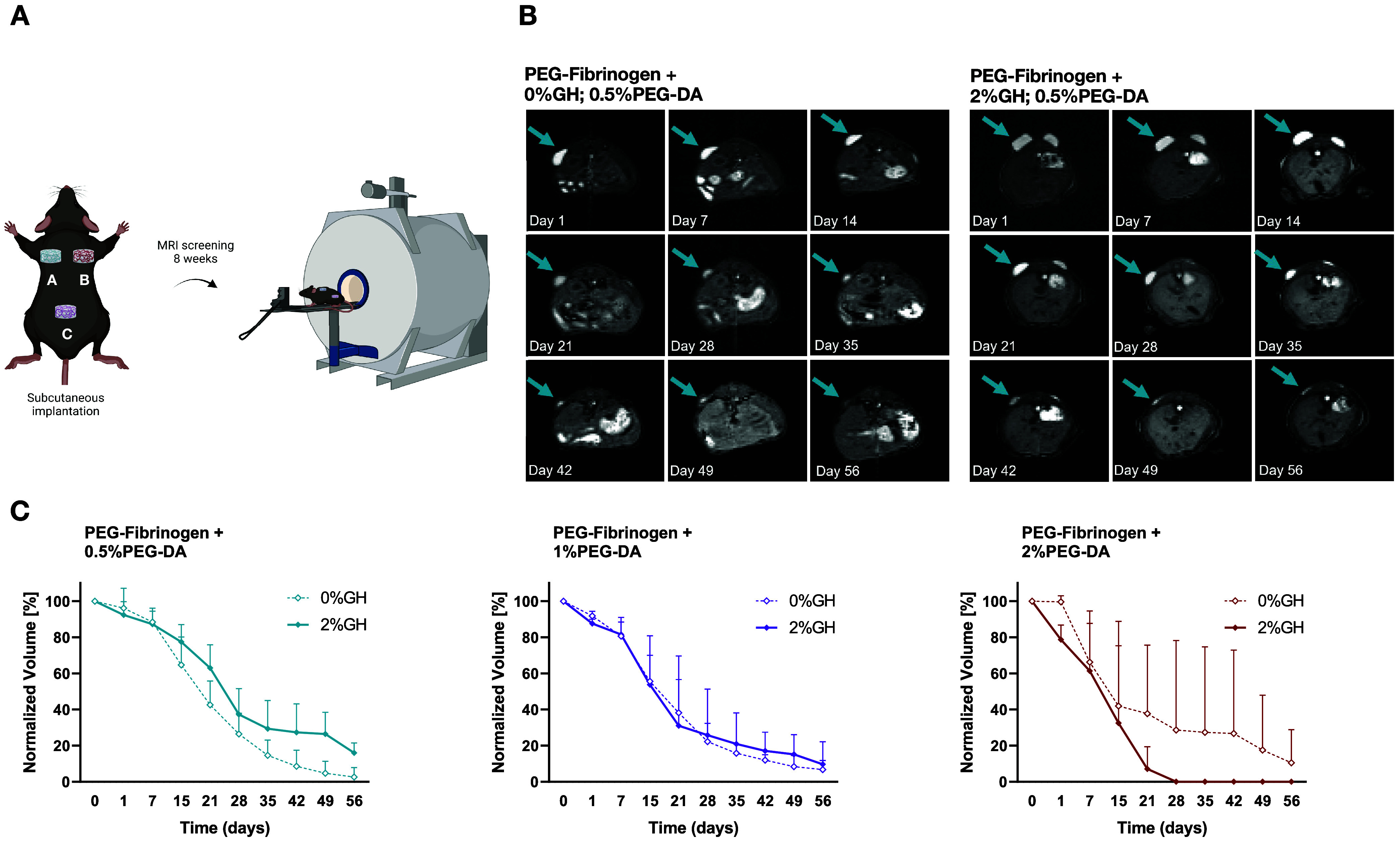
Bioresorption of IPN and single-network PF hydrogels after subcutaneous implantation in adult male C57BL/6 mice, assessed by quantitative MRI analysis. (A) The acellular hydrogel plug constructs were made with gadolinium-labeled formulations, including 8 mg/mL PF with different concentrations of PEG-DA (0.5, 1, 2%) and GH-HA (0, 2%). MR imaging was performed weekly for 8 weeks. (B) Representative images acquired with MRI of PF with 0.5% PEG-DA, with and without 2% GH-HA (additional treatments are shown in Supplementary Figure). Arrows indicate the location of the plug construct. (C) Quantitative analysis of the construct volume normalized to day 1 for all formulations tested (*n* = 4, mean ± SD).

Quantitative analysis of the implant volume after 8 weeks demonstrated a significant decrease from the initial volume (*p* < 0.05, ANOVA), indicating a progressive biodegradation of the matrix. The implant volume of most of the formulations was decreased by approximately 80 to 90% after 8 weeks. For the single-network PF material, the amount of bioresorption at the final time point was dependent on the PEG-DA composition, with higher cross-linked formulations exhibiting lower volume decreases at 8 weeks. For the IPN implants, the presence of the GH-HA either increased or decreased the amount of degradation after 8 weeks, depending on the composition of PEG-DA in the IPN. For the high PEG-DA IPN formulation, the GH-HA significantly accelerated the biodegradation of the implant, whereas with the low PEG-DA IPN formulation, the GH-HA appeared to slow down the implant degradation.

The weekly evaluation of the implant volume provided further insights into the bioresorption patterns, particularly for the IPN. A nonlinear degradation pattern was evident in all treatments, including SN and IPN formulations. For the 0.5 and 1% PEG-DA formulations with and without GH-HA, there was an initial rapid degradation phase until about 3 weeks, followed by a more gradual bioresorption over the remaining time. Interestingly, the PF implants containing GH-HA and 2% PEG-DA exhibited a different bioresorption pattern compared to all the other formulations, including formulations without the GH-HA. Specifically, the PF implants with GH and 2% PEG-DA were completely resorbed after 4 weeks, whereas all other formulations did not demonstrate such rapid resorption. It is important to note that such MRI biodegradation data is typically highly variable, particularly for PF formulations with higher concentrations of the PEG-DA cross-linker.^57–60^

### Histological Assessment

The approximate implant locations on the backs of the mice were photographed and inspected for noticeable signs of adverse tissue response to the hydrogel implants (Figures S4–S6). There were no adverse reactions to the implant that were visible on the backs of most of the treated animals, with the exception of one animal that exhibited adverse reaction to one of the hydrogel implants (mouse #1, formulation: PF+2%PEG-DA 7). This adverse reaction was still visible after 8 weeks (Figure S4). This tissue response was evaluated histologically after 8 weeks to reveal signs of foreign body reaction with a granuloma formation composed of multinucleated giant cells surrounding the implanted hydrogel (Figure S4). Histological examination by H&E staining of all other formulations revealed normal repair tissue at the end of the 8-week experiment. Representative samples consisting of skin and muscle at the approximate location of the implanted materials are shown with normal repair tissue (Figures S5 and S6). Most tissue samples had minimal or no inflammation after 8 weeks, as confirmed by H&E staining. One of the treated sites on mouse no. 7 showed signs of a fresh scab (Figure S5B).

## Discussion

The extracellular matrix (ECM) is a remarkably complex biomaterial capable of enabling intricate cellular activity including motility, morphogenesis, and remodeling—without compromising its structural stability and mechanical integrity.^61^ For example, the ECM of skin is composed of collagen and elastin fibers reinforced with proteoglycans (PGs) and glycosaminoglycans (GAGs) as well as other fibrous proteins;^62^ it has strength and resilience, yet fibroblasts can readily migrate within the matrix. In tissue engineering, hydrogel scaffolds have been extensively applied to create mimics of the ECM having similar features so cells can grow, remodel, and form mature tissues. To achieve such capabilities from synthetic surrogates of the ECM, one has to provide both structural and biofunctional features to the cells residing within the matrix.^63^ The prevailing hydrogel design in this context has been to incorporate key aspects of the natural cellular microenvironment, including proteolytically degradable cross-links, cell adhesion motifs, and bioactive factors.^64,65^ Recently, hydrogels have been designed with important mechanical features to regulate cell behavior in 3D culture, including viscoelastic properties.^39,66,67^ However, many hydrogels, particularly amorphous polymeric networks, lack important structural features that are inherent to most tissues in the body. IPN hydrogels provide an opportunity to engineer more complex cellular microenvironments that incorporate structural, viscoelastic, and biofunctional features into a single matrix.^68^

In our previous work with single-network PF hydrogels, wherein we characterized the network as amorphous with a nanometer-scale mesh size,^69^ we found that 3D MSC spreading depended upon both proteolytic degradation and the degree of covalent cross-linking of the matrix (i.e., the modulus and proteolytic resistance).^49^ Hence, we concluded that it is difficult to independently control cell spreading and modulus in the single-network PF without additional design considerations. Our previous attempt to design independent control of these two parameters involved nanostructuring the PF hydrogels with various polymeric porogens.^31,32,70,71^ In this study, we sought to use an IPN-based strategy for this purpose.^72^ Specifically, we used an IPN hydrogel with a covalent gel made from PF and a physical gel made from GH-HA. The PF provides the desired control over the modulus through covalent cross-links with additional PEG-DA, whereas the GH-HA provides structural features that can independently be used to regulate MSC morphogenesis. We achieved this design criterion based on an approach first described by Kutty et al.^34^ They produced a semi-IPN that combines an amorphous PEG-based hydrogel with HA to improve cell spreading in highly cross-linked covalent gels that would otherwise inhibit this behavior. They combined hydrolytically degradable, covalently cross-linked PEG-DA endowed with cell-adhesive RGD peptides and an enzymatically degradable HA component. They found that 0.12% (w/v) HA supported fibroblast spreading throughout the 3D PEG-DA network and that cell spreading was entirely inhibited by the addition of hyaluronidase inhibitors, leading to their conclusion that enzymatic HA degradation is the critical underlying mechanism of this morphogenesis. The results in our study show a similar enhancement in cell spreading in our IPN compared to the SN hydrogels made from PF and PEG-DA, most likely owing to the dynamic nature of the physical GH-HA network and the possibility of network imperfections in the PF+PEG caused by degraded HA.

We characterized the rheological properties of our IPNs, the cell spreading within these gels, and the in vivo biodegradation of these gels to gain further insight into their unique set of properties. By incorporating the GH-HA into the PF+PEG-DA network, we found that the IPN hydrogels retained precise control over shear storage modulus, G′, through addition of the PEG-DA cross-linker. While the GH-HA did not significantly affect the G′ of the IPN relative to the SN hydrogels, the dynamic interactions facilitated by the GH-HA substantially enhanced the viscoelastic properties of the IPN, endowing these hydrogels with a significantly higher shear loss modulus and tan delta (Table 1). Another interesting effect of GH-HA in IPN was related to the kinetics of the photopolymerization. The rheological time-sweep measurements revealed that hydrogels containing higher concentrations of PEG-DA required more time to reach their plateau G’ value, and the presence of GH-HA negated this delay without altering the final modulus values. This result suggests that the presence of GH-HA affects the kinetics of the free-radical polymerization reaction.

In our previous work with these IPNs, PF with 2% PEG-DA and 3% GH-HA produced physical gels with G′∼100 Pa and G″∼20 Pa, whereas PF with 2% PEG-DA and 5% GH-HA produced physical gels with G′∼1000 Pa and G″∼500.^23^ These results demonstrated that these IPN hydrogels containing higher concentrations of GH-HA (3–5%, w/v) can undergo rapid physical gelation independent of the covalent gelation reaction.^46^ However, the formulation used in the present study, PF with 2% PEG-DA and 2% GH-HA, was unable to form a physical hydrogel without covalent chemistry (both the G′ and G″ values were approximately 10 Pa; data not shown). This indicated that the minimal concentration required for GH-HA gelation into viscoelastic hydrogel was not reached.^53^ Therefore, the hydrogel in the current study may be better classified as a semi-IPN.^73,74^ Nevertheless, the synergistic interactions between the lower concentration of GH-HA and the cross-linked PF+PEG-DA network did provide sufficient structural versatility and viscoelasticity to significantly impact cell spreading. A better understanding of the mechanisms responsible for this is still needed and will be critical to further tailoring hydrogel formulations for the 3D culture of various cell types. In the present study, we also limited the experimental design to IPN formulations with no more than 2% PEG-DA because PF gels made with higher PEG-DA content are not fully degradable by collagenase (i.e., they are not fully degradable by proteolysis alone).^50^ Having hydrogels that are not fully biodegradable by proteolysis would most certainly affect in vivo bioresorption.

In our effort to understand the mechanism responsibile for enhanced cell morphogenesis in stiff hydrogels, we devised experiments to show that the combination of covalent cross-links and GH-HA interactions provides a more versatile structure for cell spreading, perhaps mimicking more accurately the ECM by creating a more supportive environment for hMSCs growing within. We found the PF with 0.5% PEG-DA and 2% GH-HA formulation to be particularly suitable for long-term cell viability (>90% cell viability). Although a reduction in viability was not observed with the low PEG-DA treatment (i.e., 0.5% PEG-DA), there was some statistically significant reduction in viability over time in the treatment with the highest PEG-DA concentration (i.e., 2% PEG-DA). We speculate that reduction in viability is most likely associated with confinement effects caused by cells entrapped within a PEG-DA network. We and others have shown that confinement effects within PEG-DA can be detrimental to viability over time,^75,76^ mainly because of the limited degradation of the PEG network in the time scale of our experimental design. Although our cell viabilty data does not account for differences in proliferation, the quantitative cell viability assay uses a cell count algorithm of dead cells to total cells and normalizes these data accordingly to minimize bias by cell proliferation. Nevertheless, cell proliferation should be investigated as part of future studies.

The hMSCs formed 3D cellular networks within the hydrogels, as indicated by the percent cell spread area, suggesting that the matrix supported cell growth. However, we cannot differentiate between cell spreading and cell division associated with this cell growth outcome, because we did not measure cell proliferation. Previously, we reported increased proliferation of MSCs in PF with the addition of 3% GH-HA as compared to single-network PF hydrogels.^46^ We also reported on the spreading and differentiation of MSCs in single-network PF hydrogels.^77^ Herein, we focus on the observation that the GH-HA played a crucial role in promoting cell spreading within the PF+PEG-DA formulations that would otherwise be somewhat inhibitive to cell spreading.^30,49,77^ The influence of this second network on spreading patterns was almost immediate, starting within 1 week of culture. The impact of the GH-HA on cell spreading is more pronounced as the PF matrix becomes more inhibitive through the addition of PEG-DA cross-linker. Hence, this effect is most apparent in the group with the highest concentration of PEG-DA (2%) in the PF network. In the absence of GH-HA, cells in this formulation displayed less cell spreading (lower aspect ratio and smaller percent cell spread area). Interestingly, upon inclusion of the GH-HA, all IPN formulations exhibited a similar morphogenesis pattern, as no significant differences in spreading were observed among the different PEG-DA formulations at day 21. This behavior persisted even after 3 weeks of culture (data not shown), with above 90% cell spread area, regardless of the concentration of PEG-DA added. These findings suggest that as time progressed, the cells successfully prevailed over the initial constraints posed by the more cross-linked hydrogel, leading to more extensive cell spreading. This further highlights the ability of the GH-HA to expedite cell spreading within the IPN matrix, irrespective of its modulus.^12^ Although we did limit our experimental design herein to IPN formulations with 2% PEG-DA due to considerations with proteolysis of the gels, IPNs containing even higher concentrations of PEG-DA (up to 6% w/v) were preliminarily screened for cell morphogenesis at day 1 to reveal a consistent trend (Figure S7).

Others have reported on the use of IPN and semi-IPN hydrogel systems to study the relationship between cell spreading and the hydrogel modulus. Aprile et al. cultured MSCs in alginate-collagen IPNs and investigated the effects of hydrogel modulus on 3D cell spreading and chondrogenic differentiation.^78^ They reported more spreading in soft materials (*E* = 2.5 kPa) as compared to stiffer materials (*E* = 17.5 kPa) and claimed that their IPN system enables the independent control of substrate stiffness and cell morphology in 3D culture. Vorwald et al. cocultured MSCs and endothelial cells (ECs) in fibrin-alginate IPN hydrogels to investigate the effects of structure, degradation, modulus, and mesh size on the cell morphogenesis.^79^ They found that lower-modulus hydrogels (G′ <1 kPa) supported increased cell spreading as compared to higher modulus hydrogels (G′ >2.5 kPa). They concluded that cell adhesion and hydrogel bulk stiffness can be decoupled using their IPN system. Sun et al. cultured MC3T3-E1 preosteoblast cells in an IPN of methacrylated alginate (MAA) and collagen to show that the percentage of cells spreading with the IPN was drastically increased compared to the single-network MAA gels.^38^ Crosby et al. cultured iPSC-derived endothelial progenitor cells in IPN hydrogels composed of collagen and norbornene-modified hyaluronic acid (NorHA) with a peptide cross-linker.^80^ They were able to control the mechanical properties (G′ in the range of 50 to 300 Pa) by varying the concentration and sequence, respectively, of the NorHA peptide cross-linker. Their results showed that iPSC-derived microvascular network formation was enhanced in less degradable hydrogels, and cell growth was restricted in the higher modulus hydrogels. Lou et al. used an IPN hydrogel made from HA cross-linked with dynamic covalent bonds and type I collagen to mimic the viscoelasticity and fibrillar structure of the ECM.^39^ They showed that the unique stress-relaxation behavior afforded by the IPN constituents can be fine-tuned to exhibit faster relaxation, which facilitated more cell spreading, fiber remodeling, and focal adhesion (FA) formation in the hydrogels. Wei et al. designed dynamic IPN hydrogels with gelatin and dextran, showing that these dynamic networks can increase the contractility of human endothelial colony-forming cells (hECFCs).^81^ They demonstrated a link between the viscoelastic response of their dynamic hydrogels and activation of focal adhesion kinase (FAK) as well as metalloproteinase expression, leading to an accelerated vasculogenic response in vivo. Brunel et al. describe an IPN made from both fibrillar and amorphous collagens.^37^ They showed that supramolecular structures of this IPN have in the form of fibrils that facilitate cellular interactions that help overcome the constraints of the amorphous collagen matrix. Unfortunately, it is difficult to make direct comparisons between our findings and these different studies because these IPNs behave differently from the PF–PEG-HA material.

Lee et al. worked with a semi-IPN that more closely resembles the material system described in our work. They prepared a semi-IPN composed of hydrolytically degradable PEG-DA, acrylate-PEG-GRGDS (cell-adhesive peptide), and native HA.^82^ They compared fibroblast cell spreading in their semi-IPNs, SN PEG-DA hydrogels, and SN methacrylated-HA (MeHA) hydrogels. The PEG-DA/HA semi-IPNs were able to support cell spreading at relatively high levels of mechanical properties (*E* ∼ 10 kPa) compared to those of PEG-DA and MeHA hydrogels. They showed that the cell spreading in the PEG-DA/HA semi-IPNs was hindered by the increased amounts of PEG-DA cross-linking of the system and somewhat dependent on the molecular weight of the HA. They concluded that the underlying mechanism responsible for the enhancement in cell spreading in their semi-IPNs (when compared to SN PEG-DA hydrogels) was a polymerization-induced phase separation that resulted in HA-enriched defects within the covalent PEG-DA network structure. Our findings could be explained by the mechanism described by Lee et al. Specifically, we speculate that HA-enriched heterogeneity in the PF+PEG-DA hydrogels could serve to create network imperfections that better facilitate cell spreading within the IPN. Moreover, cell-mediated enzymatic degradation of HA, as previously reported by Kutty et al., namely, by hyaluronidases, can break down HA into smaller fragments and produce larger imperfections. This enzymatic degradation of HA within the IPN hydrogel over time creates a dynamic microenvironment that facilitates cell spreading and proliferation. Additionally, the HA that undergoes enzymatic degradation generates smaller fragments that can interact with cell surface receptors and influence the cell behavior. These fragments can promote cell adhesion, migration, and proliferation, thereby enhancing the overall cellular response within the hydrogel.

The in vivo biodegradation experiments were performed to provide further insight into how the unique IPN network structure affects the resorption of these PF-based materials. The biodegradation of the hydrogels was analyzed weekly by MRI over an 8-week period, demonstrating a gradual decrease in implant volume. As expected, the SN PF hydrogels exhibited more resistance to degradation over time with increasing concentrations of PEG-DA. This MRI data is completely consistent with what is known about the bioresorption patterns of these amorphous materials, namely, that surface degradation is hindered by the proteolytic resistance of the material as dictated by the cross-linking density (i.e., PEG-DA content).^58,59^ However, this pattern was completely reversed in the IPN hydrogels, where inclusion of GH-HA led to faster degradation rates with increasing concentrations of PEG-DA. What is unexpected about the MRI data from the IPN treatment is the apparent acceleration of bioresorption with increasing levels of PEG-DA; full degradation of the GH-HA-PF+2% PEG-DA implant was observed after only 4 weeks as compared to >8 weeks for the other IPN treatments. Also apparent from these data is that the presence of GH-HA itself does not accelerate the breakdown of the PF materials as can be observed by comparing the slower bioresorption kinetics of the IPN containing 0.5% PEG-DA to the relatively faster bioresorption of the SN PF+0.5% PEG-DA. Evidently, the interaction between the GH-HA and the additional PEG-DA cross-linking reduces the resistance of the IPN to proteolytic breakdown in vivo. Consensually, the shear storage modulus of the GH-HA-PF+2% PEG-DA was slightly lower when compared to the SN PF+2% PEG-DA, whereas the storage modulus tended to be higher for the 0.5 and 1% GH-PF+PEG-DA formulations when compared to their respective SN PF formulations. Although the differences within each formulation did not prove to be statistically significant, the trend reversal for the G′ of the 2% formulations stood out and could possibly provide further insights as to the mechanism for the accelerated in vivo degradation of this composition. Importantly, the MRI data cannot differentiate between surface erosion-mediated biodegradation, bulk biodegradation, and hydrogel swelling; therefore, we are unable to exclude the possibility that more than one mechanism is involved in the volumetric changes that were observed for the different treatments.

An unexpected result in this study was the reduced modulus of the 2% PEG-DA+GH hydrogels. Although one would expect the GH to contribute to cross-linking (and increased modulus) of the PF+PEG-DA hydrogels, we observe a reduction in modulus, but only with the 2% PEG-DA+GH formulation (Figure 3C). This observation is likely associated with a macromolecular phenomenon that occurs during assembly of the 2% PEG-DA+GH IPN formulation. We speculate that the less hydrophilic fibrinogen and the highly hydrophilic PEG-DA additive in solution and at these concentrations undergo complex macromolecular interactions. Specifically, fibrinogen and PEG-DA compete for solvent (i.e., water) in the precursor state, altering the PF’s macromolecular arrangements prior to photo-cross-linking. We previously reported on the PF’s macromolecular organization using small-angle light scattering, where we showed that the PEGylated fibrinogen undergoes some aggregation prior to photochemistry resulting in larger macromolecular networks that likely shield some of the PEG-DA reactive groups from the radical polymerization reaction.^69^ In a subsequent study, we showed that this organization was highly influenced by the composition and properties of the synthetic polymer in the system as it interacts with the fibrinogen polypeptide.^83^ Consequently, in the pure PF+PEG-DA formulation, adding more synthetic polymer also increases the cross-linking density, introducing more functional acrylate groups. This in turn counterbalances the steric effects and results in an overall increase in the modulus of the PF+2% PEG-DA formulation. However, with the introduction of a third polymer (i.e., GH-HA) that is also competing for solvent, this balance could be further confounded. We speculate that the presence of the 2% PEG-DA and 2% GH affects the macromolecular arrangement of PF to such an extent that the result is a substantial agglutination of PF due to a shifting balance between protein-bound, HA-bound, and PEG-bound water. Hence, the radical polymerization and subsequent cross-linking efficiency are reduced beyond any benefit provided by the GH-HA cross-linking, thereby causing the observed reduction in the presence of GH in the 2% PEG-DA PF formulation. This reduced cross-linking can also alter the in vivo biodegradation of this formulation, thereby providing a possible explanation for the more rapid in vivo resorption of this formulation.

In terms of in vivo biocompatibility, all implant compositions proved to be similar in that they did not elicit an adverse tissue response, as indicated by histological assessments after 8 weeks. It is important to note that further investigations are required to fully understand the intricacies of this cell-mediated enzymatic degradation process and its precise effects on both the in vivo resorption and the cellular responses within the hydrogel. Nonetheless, the current findings provide a good foundation for future studies that aim to design biomaterials with control over modulus, in vivo biodegradation, and 3D cell morphogenesis.

## Conclusions

In the current study, we set out to develop a hydrogel matrix for 3D MSC culture, with independent control over modulus and morphogenesis. Building upon our earlier work with IPNs made from PF and GH-HA that revealed unique 3D spreading patterns within these materials, we investigated how the structural versatility afforded by GH-HA affected MSC viability, 3D cell spreading, and in vivo biodegradation kinetics. From our results, we concluded that the combination of the GH-HA and PF endowed these IPN materials with more viscoelasticity yet did not alter the ability to precisely control the hydrogel shear storage modulus using additional PEG-DA cross-linker. We also concluded that the PF–PEG-HA IPN can facilitate more cell spreading independent of the material modulus when compared to single-network PF hydrogels. Finally, we concluded that increasing PEG-DA cross-linking of the IPN accelerates the in vivo bioresorption. Although all of these unique properties have been demonstrated only within the limits of the formulations tested, we believe that they are somehow linked to an enhanced structural versatility of our IPN, where dynamic network imperfections associated with the GH-HA help augment cell morphogenesis and cell-mediated proteolytic degradation. As we pursue a better understanding of these causative factors, we also seek to apply these insights toward designing materials with independent control over viscoelastic properties, cellular morphogenesis, and in vivo biodegradation. These features can be very useful to mimic native tissue structure and function using a materials engineering design strategy.

## Supplementary Material





## Notes

Additional experimental results, calibrations, MRI images, and histology, including photographs of gross appearance of implant site. The Supporting Information is available free of charge at https://pubs.acs.org/doi/10.1021/acsbiomaterials.5c00980.

Additional experimental results, calibrations, MRI images, and histology, including photographs of gross appearance of implant site (PDF)
hMSC viability of PEG-Fibrinogen + 2% GH hydrogels with increasing PEG-DA content (0.5–6%) on day 1 of 3D culture (Figure S1); MRI relaxivity calibration curves for PF-GdDTPA within PEG-DA hydrogels (Figure S2); In vivo MR imaging of PEG-Fibrinogen + 1% PEG-DA and 2%PEG-DA hydrogels with and without GH over time (Figure S3); Histological analysis of PEG-Fibrinogen + 0% GH hydrogels with varying DA concentrations (Figure S4); Histological analysis of PEG-Fibrinogen + 2% GH hydrogels with varying DA concentrations (Figure S5); Histological analysis of PEG-Fibrinogen + 2% GH hydrogels with varying DA concentrations (Figure S6) (PDF)

## △

Department of Bioengineering, University of Pennsylvania, Philadelphia, PA 19104, United States
