# Supplementary material for: Interpenetrating Polymer Network Hydrogel Composition Alters Encapsulated MSC Spreading and In Vivo Degradation Behavior
Source: ACS Biomater Sci Eng. 2025 Aug 4;11(9):5586–99. doi: 10.1021/acsbiomaterials.5c00980 (PMC12421499; doi:10.1021/acsbiomaterials.5c00980)

Interpenetrating Polymer Network Hydrogel Composition Alters Encapsulated MSC Spreading and In Vivo Degradation Behavior

Liaura Ifergan-Azriel[1], Orit Bar-Am[1], Galit Saar[2], Talia Cohen[2], Claudia Loebel[3], Jason A. Burdick[4], Dror Seliktar[1*]

[1] The Faculty of Biomedical Engineering, Technion-Israel Institute of Technology, Haifa, Israel
[2] The Bruce Rappaport Faculty of Medicine, Technion–Israel Institute of Technology, Haifa Israel
[3] Materials Science & Biomedical Engineering Department, University of Michigan, Ann Arbor, Michigan, USA
[4] BioFrontiers Institute and Department of Chemical and Biological Engineering, University of Colorado, Boulder, Colorado, USA

Supporting Information for Publication

Example of object detection and morphological calculation using regionprops function in matlab

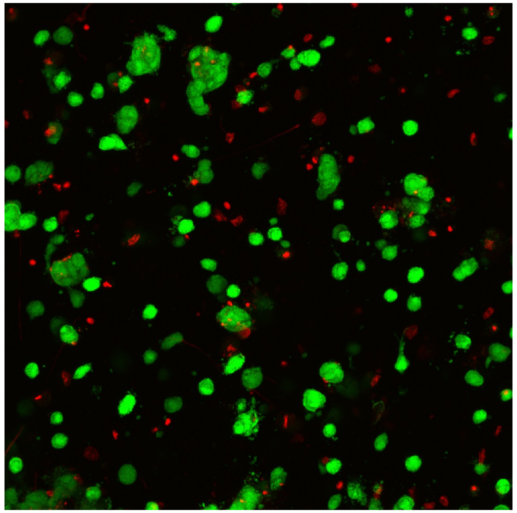

Original Picture (Merged)
Calcein-AM (green)
Ethidium (red)

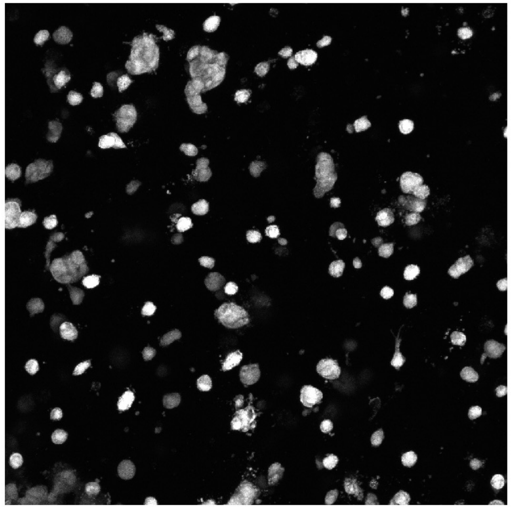

Split green channel
Binarization
Contrast Enhancement
Noise Removing

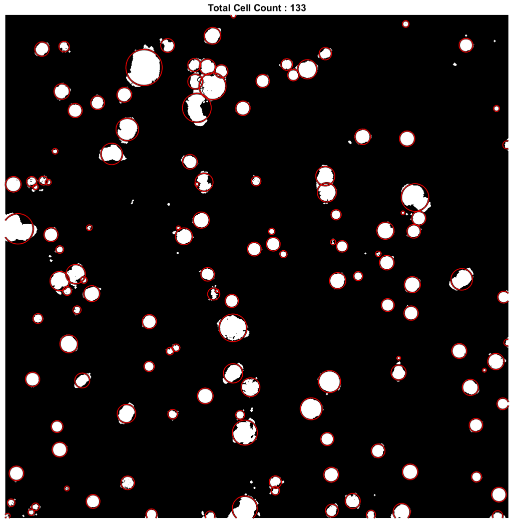

Thresholding and Watershed segmentation

Defining properties of each object (elipse) using "regionprops" and calculate the aspect ratio :

$$aspect\ ratio = \frac{Major\ Axis\ Length}{Minor\ Axis\ Length}$$

Upload results of aspect ratio of each object in each image to an excel spreadsheet for image analysis.

Supplementary Figure S2

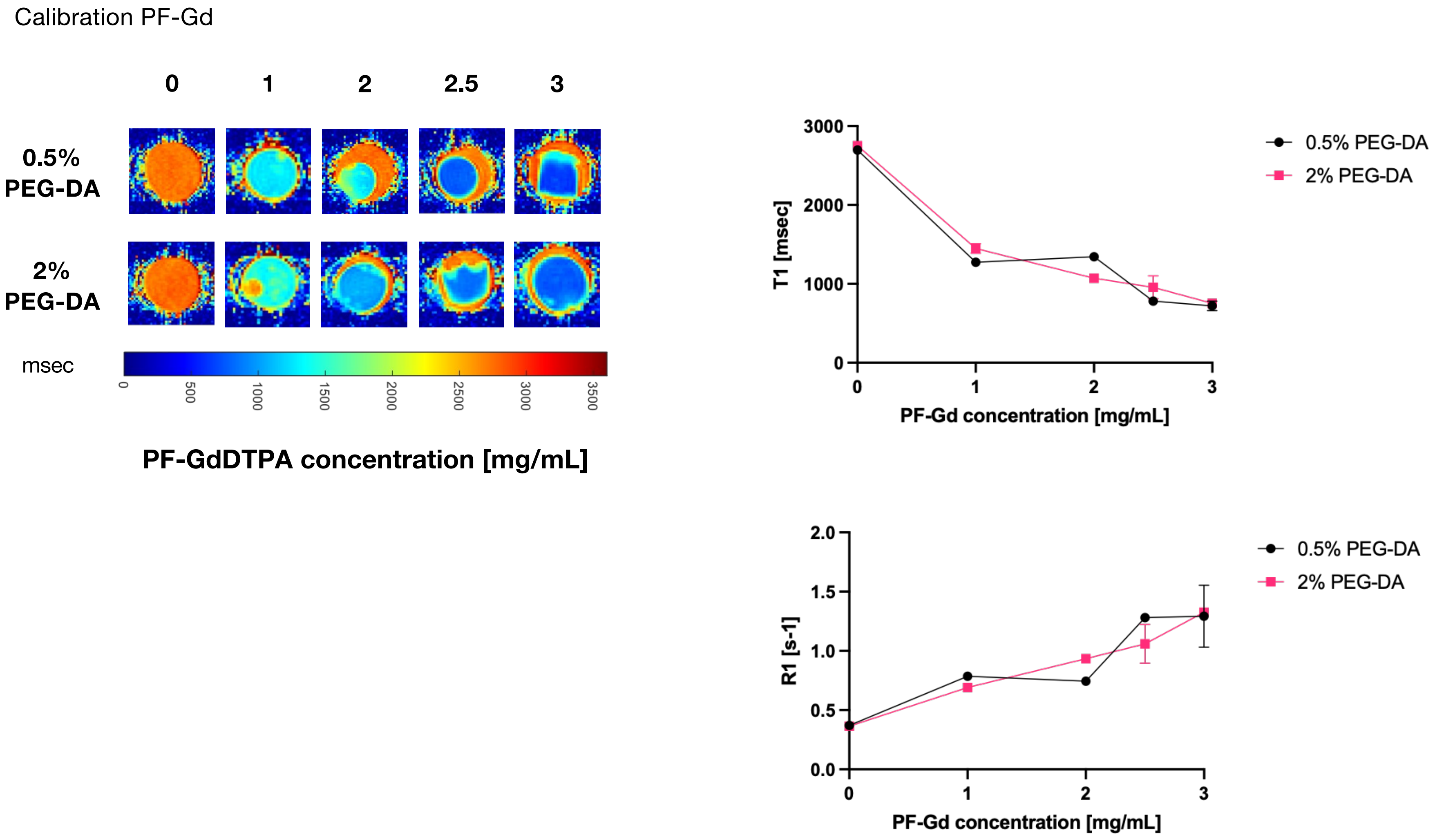

Supplementary Figure S3

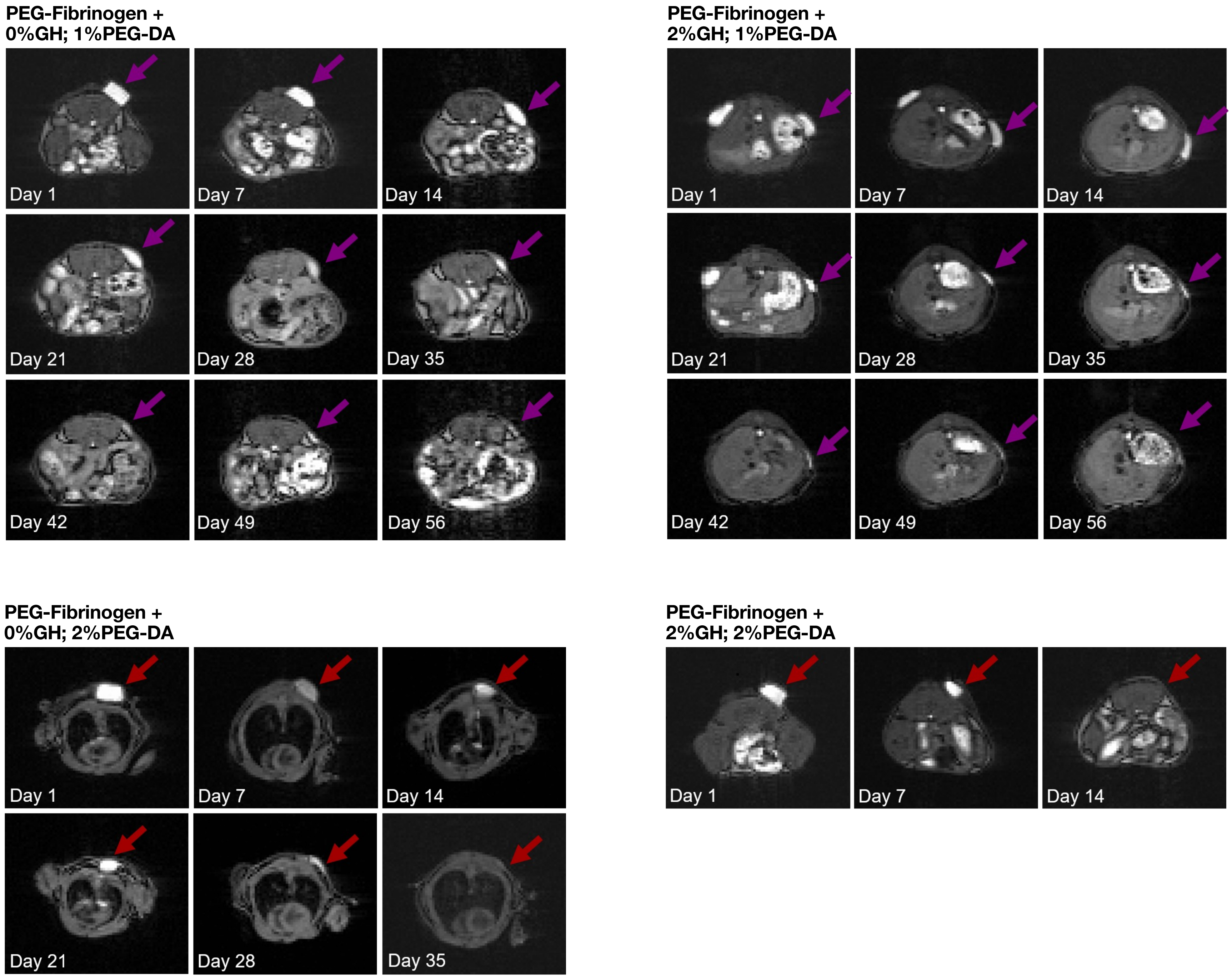

PEG-Fibrinogen + 0%GH
Mouse #1

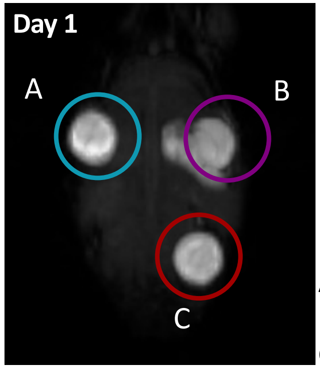

A: 0.5%DA
B: 1% DA
C: 2% DA

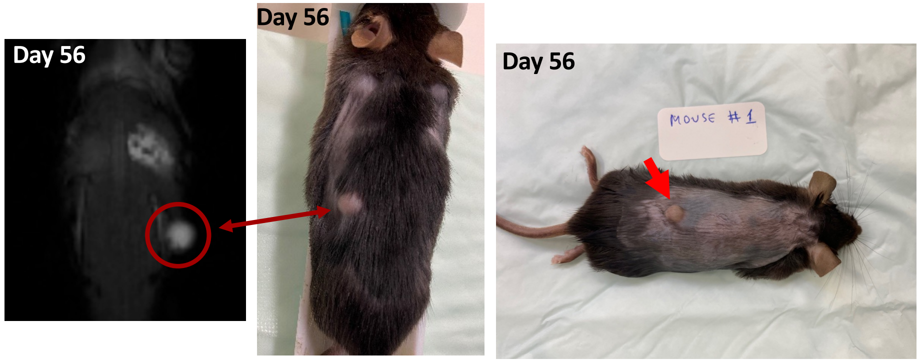

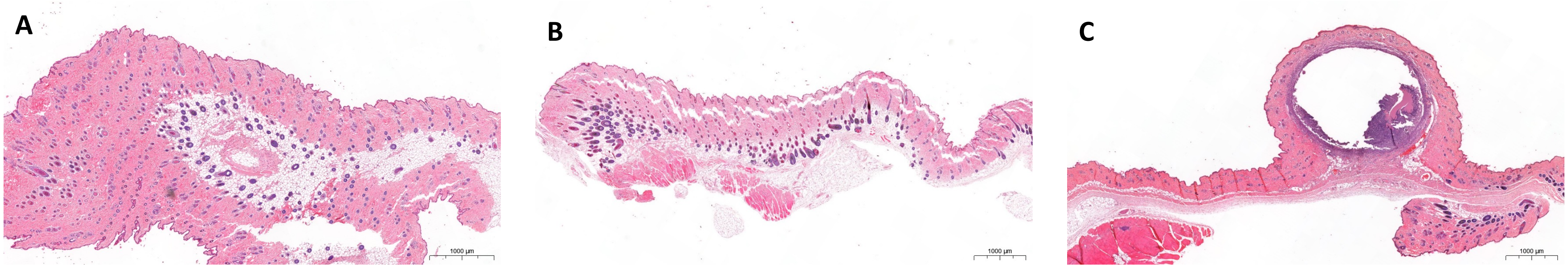

PEG-Fibrinogen + 2%GH
Mouse #7

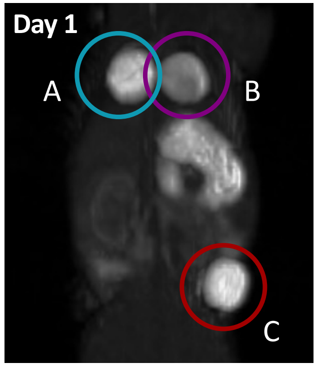

A: 0.5%DA
B: 1% DA
C: 2% DA

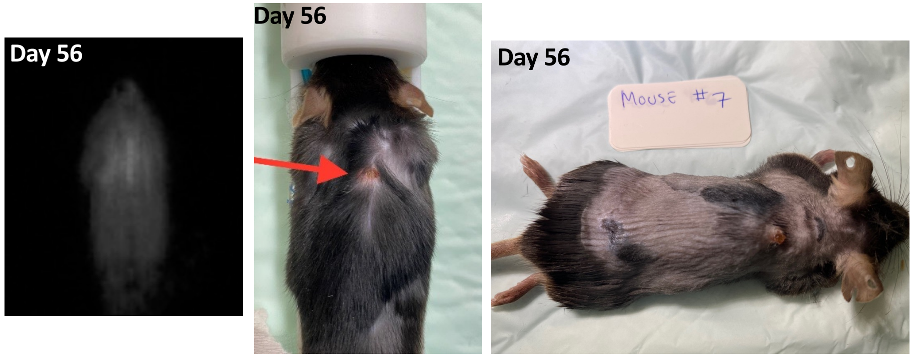

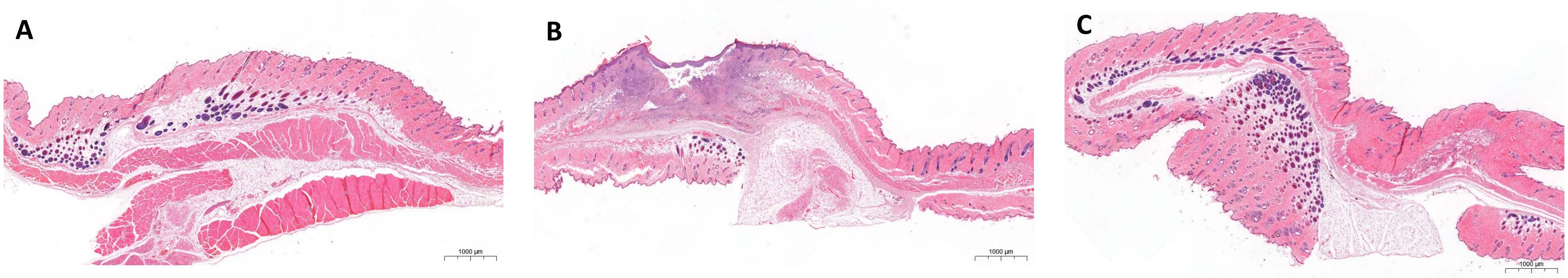

PEG-Fibrinogen + 2%GH
Mouse #5

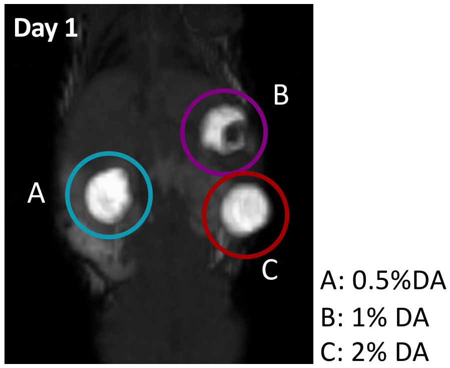

A: 0.5%DA
B: 1% DA
C: 2% DA

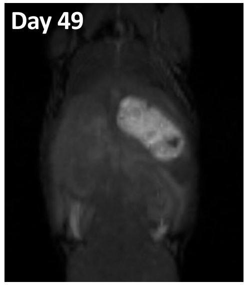

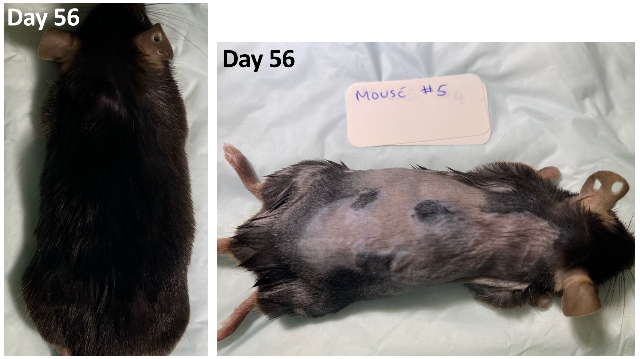

A

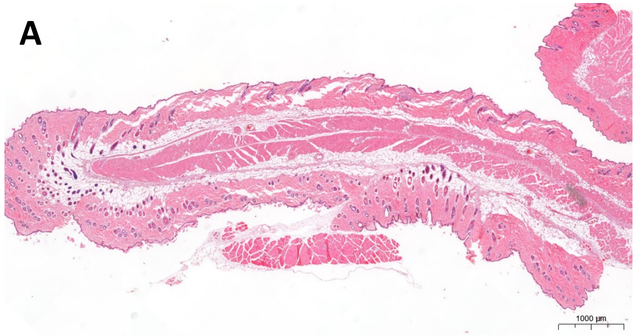

B

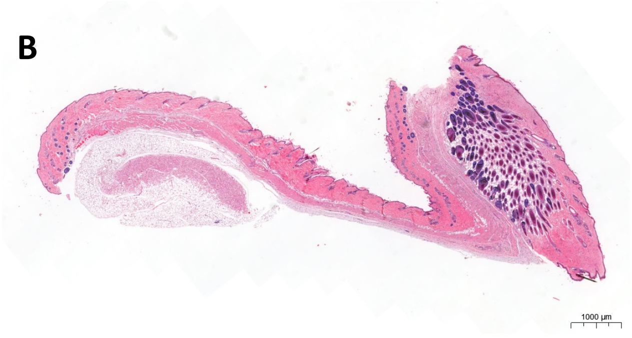

C

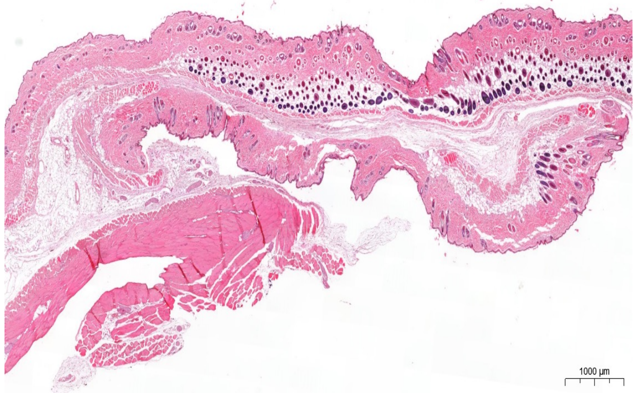

Supplementary Figure S7

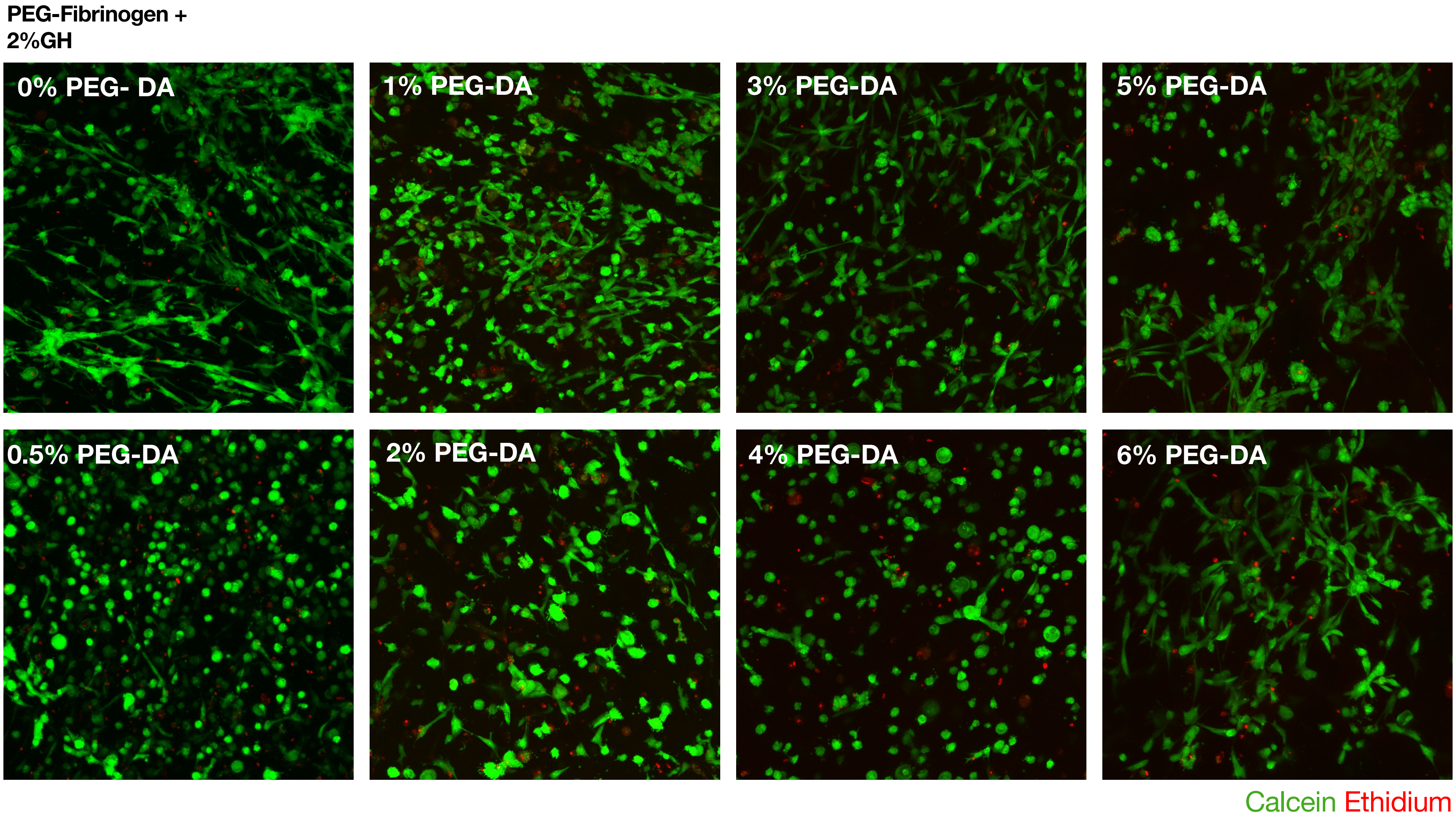

Supplement: Supplementary file 1 [file ab5c00980_si_001.pdf]
