# Supplementary material for: Interpenetrating Polymer Network Hydrogel Composition Alters Encapsulated MSC Spreading and In Vivo Degradation Behavior
Source: ACS Biomater Sci Eng. 2025 Aug 4;11(9):5586–99. doi: 10.1021/acsbiomaterials.5c00980 (PMC12421499; doi:10.1021/acsbiomaterials.5c00980)
Supplement: Supplementary file 2 [file ab5c00980_si_002.pdf]

Supplementary Figure Legends

Supplementary Figure S1. *hMSC viability of PEG-Fibrinogen + 2% GH hydrogels with increasing PEG-DA content (0.5%-6%) on day 1 of 3D culture.* Live/dead assay of encapsulated cells in PEG-Fibrinogen |(8 mg/mL) + 2% GH hydrogel constructs with increasing concentrations of PEG-DA (ranging from 0% to 6% DA). Live cells are stained with Calcein (green), while dead cells are stained with Ethidium homodimer (red). The hMCs were three-dimensionally cultured in growth (self-renewal) medium for 24 hours. An increase in DA concentration shows varying impacts on cell viability and morphology, with lower DA concentrations exhibiting more spherical morphologies and higher viability, while higher DA concentrations (4%–6%) show more elongated cell structures and increased cell death.

Supplementary Figure S2. *MRI relaxivity calibration curves for PF-GdDTPA within PEG-DA hydrogels.* MR images (left) and corresponding quantitative plots (right) of T1 relaxation time (top) and R1 relaxation rate (bottom) as a function of PF-GdDTPA concentration (0-3 mg/mL) in 0.5% and 2% PEG-DA hydrogels. Color bar indicates T1 relaxation time in milliseconds (msec). A linear correlation was found between R1 values and the PF-GdDTPA concentration based on the in vitro MRI calibration, a PF-GdDTPA concentration of 2.5 mg/ml was chosen for all the in vivo experiments because this concentration of the Gd-labelled materials showed T1 value allowing for distinguishing between the Gd-labelled hydrogel constructs and the surrounding aqueous environment.

Supplementary Figure S3. *In vivo MR imaging of PEG-Fibrinogen + 1% PEG-DA and 2%PEG-DA hydrogels with and without GH over time*. MR images of mice implanted with PEG-Fibrinogen + 1% PEG-DA or 2%PEG-DA hydrogels either without GH (left panel) or with 2% GH (right panel), taken at days 1, 7, 14, 21, 28, 35, 42, 49, and 56. Arrows point to the hydrogel implants (n=4 per group).

Supplementary Figure S4. *Histological analysis of PEG-Fibrinogen + 0% GH hydrogels with varying DA concentrations*. Representative images of H&E staining of

subcutaneous samples consisting of skin, implant, and muscle excised from mouse #1 at the locations of the implantation site after 8 weeks. The implants including three formations: (A) PF with 0.5% PEG-DA; (B) PF with 1% PEG-DA; (C) PF with 2% PEG-DA. In A and B, the images show a complete resorption of the hydrogel implant. In C, the image shows a chronic inflammatory response surrounding the implant, with remnants of hydrogel surrounded by immune cells. The MR image at day 1 and day 56, as well as the gross observation image of the animal on day 56 are shown for reference.

Supplementary Figure S5. *Histological analysis of PEG-Fibrinogen + 2% GH hydrogels with varying DA concentrations*. Representative images of H&E staining of subcutaneous samples consisting of skin, implant, and muscle excised from mouse #7 at the locations of the implantation site after 8 weeks. The implants including three formations: (A) IPN with 0.5% PEG-DA and 2% GH-HA; (B) IPN with 1% PEG-DA and 2% GH-HA; (C) IPN with 2% PEG-DA and 2% GH-HA. In A and C, the images show a complete resorption of the hydrogel implant. In B, the image shows a fresh scab near the implant location, with immune cells present. The MR image at day 1 and day 56, as well as the gross observation image of the animal on day 56 are shown for reference.

Supplementary Figure S6. *Histological analysis of PEG-Fibrinogen + 2% GH hydrogels with varying DA concentrations*. Representative images of H&E staining of subcutaneous samples consisting of skin, implant, and muscle excised from mouse #5 at the locations of the implantation site after 8 weeks. The implants including three formations: (A) IPN with 0.5% PEG-DA and 2% GH-HA; (B) IPN with 1% PEG-DA and 2% GH-HA; (C) IPN with 2% PEG-DA and 2% GH-HA. In A, B and C, the images show a complete resorption of the hydrogel implant. The MR image at day 1 and day 56, as well as the gross observation image of the animal on day 56 are shown for reference.
